# Arthrite tuberculeuse sur prothèse

**DOI:** 10.11604/pamj.2020.37.130.21360

**Published:** 2020-10-06

**Authors:** Faten Limaïem

**Affiliations:** 1 Université de Tunis El Manar, Faculté de Médecine de Tunis, Tunis, Tunisie

**Keywords:** Arthrite, tuberculose, prothèse, Arthritis, tuberculosis, prosthesis

## Abstract

Tuberculosis arthritis on prosthesis is mainly the consequence of a local reactivation of a tuberculosis infection. The clinical characteristics are broadly identical to those of other infections on prostheses except that the clinical course is often chronic, with sometimes repeated loosening when the infection has been overlooked and the presence of cold abscesses and fistulization at the skin is not uncommon. Fistulas can become infected with a common germ, which can delay the diagnosis of tuberculosis. This is the case of a 51-year-old man operated on in 2001 for a total hip prosthesis, in whom the radiological check-up revealed a mechanical loosening of the prosthesis. A surgical re-intervention was decided for this patient. Intraoperatively, rice-like granules were discovered next to the acetabulum. Histological examination of the periprosthetic samples taken showed that the rice-like granules responded to coalescent nodular formations surrounded by dense collagenous fibrosis and centered by an eosinophilic acellular amorphous necrosis. Giant scattered Langhans cells were focally noted. The final pathological diagnosis was that of caseo-fibrous tuberculosis. The patient received anti-tuberculosis treatment. He is currently undergoing orthopedic consultation. The clinician must think about tuberculous arthritis in case of mechanical loosening of a prosthesis. It is therefore essential to take microbiological samples, to look for BAAR with culture in a specific environment in search of mycobacteria. The pathological examination confirms the diagnosis with certainty.

## Image in medicine

L´arthrite tuberculeuse sur prothèse est majoritairement la conséquence d´une réactivation locale d´une infection tuberculeuse. Les caractéristiques cliniques sont globalement identiques à celles des autres infections sur prothèses si ce n´est que le tableau est souvent chronique, avec parfois des descellements répétés lorsque l´infection a été méconnue et la présence d´abcès froids et d´une fistulisation à la peau n´est pas rare. Les fistules peuvent se surinfecter à un germe banal, pouvant alors retarder le diagnostic de tuberculose. Il s'agit d'un homme âgé de 51 ans opéré en 2001 pour prothèse totale de la hanche, chez qui le bilan radiologique de contrôle a objectivé un descellement mécanique de la prothèse. Une ré-intervention chirurgicale fut décidée pour ce patient. En peropératoire, des granules riziformes ont été découverts en regard des bourrelets cotyloïdiens. L'examen histologique des prélèvements péri-prothétiques effectués a montré que les granules riziformes répondaient à des formations nodulaires coalescentes cernées par une fibrose collagène dense et centrées par une nécrose éosinophile grumeleuse acellulaire amorphe. Des cellules géantes éparses de type Langhans étaient par endroit notées. Le diagnostic retenu était celui d'une tuberculose caséo-fibreuse. Le patient a bénéficié d'un traitement anti-tuberculeux. Il est actuellement suivi à la consultation externe d'orthopédie. Le clinicien doit savoir penser à une arthrite tuberculeuse devant tout descellement mécanique d´une prothèse. Il est alors indispensable de faire des prélèvements microbiologiques, à la recherche de BAAR avec culture en milieu spécifique à la recherche de mycobactérie. L´examen anatomopathologique permet de confirmer avec certitude le diagnostic.

**Figure 1:**
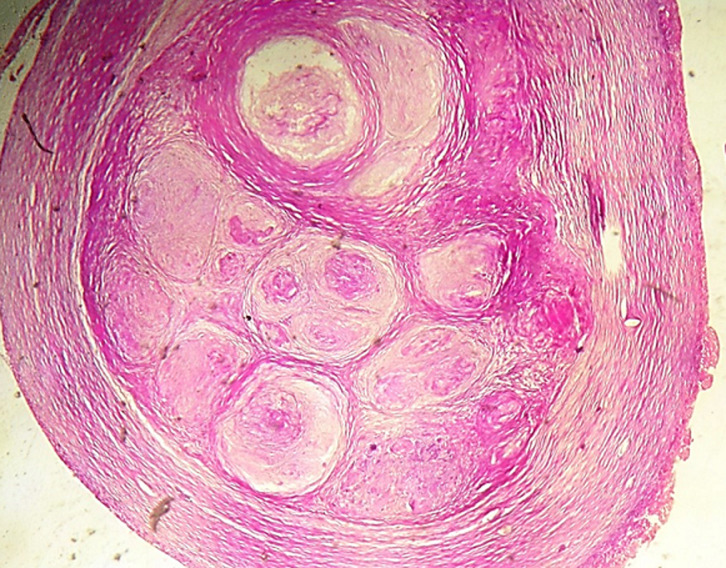
formations nodulaires coalescentes cernées par une fibrose collagène dense et centrées par une nécrose éosinophile grumeleuse acellulaire amorphe (nécrose caséeuse) (hématoxyline et éosine × 100)

